# Sweet enhancers of polymerase chain reaction

**DOI:** 10.1371/journal.pone.0311939

**Published:** 2024-10-29

**Authors:** Binghua Xie, Jia Chen, Zhounan Wang, Qiao Yin, Zhong-Min Dai

**Affiliations:** Key Laboratory of Organ Development and Regeneration of Zhejiang Province, and College of Life and Environmental Sciences, Hangzhou Normal University, Hangzhou, Zhejiang, China; University of Florida Institute of Food and Agricultural Sciences, UNITED STATES OF AMERICA

## Abstract

Although faster and powerful, polymerase chain reaction (PCR) often failed to amplify targets efficiently. Numerous PCR enhancers have been used to increase the amplification efficiency of difficult DNA targets. However, there is no systematic comparison of their effects in normal and difficult PCR conditions. In this paper, we have selected nine different PCR enhancers that can promote the PCR amplification efficiency. We have compared their effect in Taq DNA polymerase thermostability, inhibitor resistance, and amplification of various DNA targets. Although the PCR enhancers more or less reduced the amplification efficiency of DNA fragments with moderate GC-content, they were able to improve the amplification efficiency and specificity of GC-rich fragments. Betaine outperformed the other enhancers in amplification of GC-rich DNA fragments, thermostabilizing Taq DNA polymerase, and inhibitor tolerance. Sucrose and trehalose showed similar effect in thermostabilizing Taq DNA polymerase and inhibitor tolerance, while they showed mildest inhibitory effect on normal PCR. For GC-rich region-containing long DNA fragment amplification, 1 M betaine, 0.5 M betaine + 0.2 M sucrose, or 1 M betaine + 0.1 M sucrose can be used to effectively promote the amplification, while keep their negative effect in amplification of normal fragment to a minimal level.

## Introduction

Polymerase chain reaction (PCR) is a fast and powerful method in amplifying target DNA fragment, which has rapidly become one of the most widely used techniques in basic bioscience research, diagnostics and forensic science [1–3]. Numerous thermostable DNA polymerases with distinct properties were identified and used to improve PCR performance and extend PCR application [4, 5].

PCR tends to be failed in amplification of difficult DNA targets with properties such as very long, GC-rich, have stable secondary structure, and containing repeated sequences. The amplification efficiency of PCR is sensitive to inhibitors such as blood, heparin, humic acids, polyphenols and polysaccharides [6]. A few PCR enhancers, such as dimethyl sulfoxide (DMSO), betaine, glycerol, formamide, ethylene glycol (EG), 1,2-propanediol (1,2-PG), trehalose, and sucrose, commonly referred to as GC enhancers, have been successfully used to increase the amplification efficiency of difficult DNA targets [7–17]. Most of the PCR enhancers improve the amplification efficiency by lowering the melting temperature (Tm) of DNA, thus ensure thorough denaturation of templates as well as prevent stable secondary structure formation. Besides lowering the melting temperature of DNA duplex, PCR enhancers such as trehalose and betaine also thermal stabilizing the DNA polymerases [13]. It is interesting that trehalose, betaine, formamide, and 1,2-PG also enhanced the PCR efficiency in the presence of PCR inhibitors such as blood and heparin [6, 12, 14, 17].

While effective in enhancing difficult DNA target amplification, PCR enhancers may inhibit PCR efficiency in some instances. For examples, PCR enhancers like formamide and DMSO thermal destabilized enzymes, therefore greatly inhibit PCR when used at high concentrations [12, 18]. Trehalose and betaine also lower the efficiency of PCR when used at high concentrations [13]. And previous study showed that betaine, formamide, and 1,2-PG decreased the polymerase extension rates with increasing concentrations [18]. To what extent these enhancers may negatively influence PCR remains elusive.

Here we tested different PCR enhancers at various conditions to compare their effect in enzyme stability, inhibitor resistance, and amplification of difficult or easy DNA targets. We found that sucrose and betaine are promising PCR enhancers that greatly improved the amplification efficiency of difficult DNA targets with little negative effect on easy-to-amplify targets.

## Materials and methods

### Prepare of PCR enhancers

1,2 propylene glycol (1,2-PG), 1,3 propylene glycol (1,3-PG), dimethyl sulfoxide (DMSO), ethylene glycol (EG), formamide, glycerol, betaine, trehalose and sucrose were purchased from Sangon Biotech. The liquid forms of 1,2-PG, 1,3-PG, DMSO, EG and formamide were directly added to the PCR reaction mixture to various concentrations (Table 1), as indicated by their volume ratios (v/v). Glycerol, a viscous liquid, was first diluted to 50% (v/v) by sterile deionized water before being added to the PCR reaction mixture at the desired concentrations (Table 1). Betaine, trehalose and sucrose were prepared as stock solutions in sterile deionized water with molarities (M) of 5 M, 1 M, and 1 M, respectively, and were used at the final concentrations listed in Table 1.

**Table 1:** The result of different concentrations enhancers for the amplification efficiency of moderate GC and GC-rich fragments by real-time PCR.

Enhancer	Concentration	53.8% GC		68.0% GC		78.4% GC	
		(moderate)		(high)		(super high)	
		
	(v/v or M)	Ct±SEM	Tm	Ct±SEM	Tm	Ct±SEM	Tm^a^
Control		15.84±0.05	85.6	15.48±0.22	89.6	32.17±0.25	94.6
Dimethyl Sulfoxide (DMSO)	2.5%	16.21±0.04	83.7	15.26±0.06	87.1	17.48±0.11	92.7
	5%	16.68±0.01	82.0	15.72±0.03	85.5	17.90±0.05	91.3
	10%	18.78±0.04	78.8	17.15±0.07	82.3	21.15±0.07	88.0
Formamide	2.5%	16.28±0.06	83.9	15.11±0.03	87.5	15.91±0.05	92.8
	5%	18.08±0.07	82.1	15.44±0.03	86.0	16.32±0.05	91.5
	10%	ND	ND	ND	ND	ND	88.2
Ethylene Glycol (EG)	2.5%	16.04±0.01	84.3	15.07±0.01	87.7	16.95±0.04	92.8
	5%	16.28±0.06	83.1	15.27±0.08	86.5	17.24±0.04	91.9
	10%	17.21±0.03	80.8	15.86±0.06	84.2	18.15±0.03	89.9
Glycerol	2.5%	16.05±0.04	84.6	15.22±0.12	88.7	16.98±0.21	93.6
	5%	16.13±0.01	83.7	15.16±0.04	87.2	16.89±0.12	92.7
	10%	16.49±0.09	82.9	15.44±0.07	85.2	17.18±0.08	91.0
1,2 Propylene Glycol (1,2-PG)	2.5%	16.14±0.06	83.7	15.18±0.10	87.2	17.09±0.12	93.0
	5%	16.44±0.12	82.2	15.45±0.03	85.5	17.37±0.08	91.8
	10%	18.56±0.30	78.8	16.96±0.07	82.4	20.41±0.15	88.2
1,3 Propylene Glycol (1,3-PG)	2.5%	16.04±0.16	84.0	15.10±0.04	87.5	16.99±0.11	93.0
	5%	16.35±0.04	82.8	15.35±0.04	86.1	17.26±0.05	92.2
	10%	17.36±0.03	80.2	16.16±0.08	83.7	19.40±0.13	89.6
Sucrose	0.1 M	15.84±0.05	85.1	15.25±0.05	89.4	23.44±0.10	94.0
	0.2 M	16.01±0.03	84.5	15.09±0.10	87.9	17.20±0.47	93.6
	0.4 M	16.39±0.09	83.5	15.03±0.04	87.0	16.67±0.08	92.7
Trehalose	0.1 M	15.86±0.00	85.1	15.11±0.05	89.4	20.22±0.64	94.0
	0.2 M	16.06±0.02	84.5	15.01±0.07	87.8	17.82±0.27	93.5
	0.4 M	16.43±0.16	83.5	15.15±0.08	86.9	16.91±0.14	92.5
Betaine	0.5 M	16.03±0.03	84.6	15.08±0.10	87.9	16.97±0.34	93.3
	1 M	16.51±0.06	83.5	15.19±0.05	86.6	16.19±0.12	92.0
	2 M	19.85±0.16	81.1	17.55±0.11	83.6	19.21±0.23	88.3

Different PCR enhancers were used at various concentrations to test their effects in amplification of moderate (53.8% GC), high (68.0% GC) and super high GC-content (78.4% GC) fragments. Ct: threshold cycle. Tm: melting temperature. ND: not determined because of low amplification efficiency.

a: The amplification of the super high GC fragment often results in products with double or multiple melting peaks, especially for those without or with low concentrations of PCR enhancers. Specific PCR products with 1 M betaine as PCR enhancer were purified, remixed with FastSYBR Mixture and PCR enhancers to measure their Tm values.

### Real-time PCR

Real-time PCR was conducted using the FastSYBR Mixture (CWBio CW0955) on a Bio-Rad CFX96 Real-Time PCR Detection System. The reaction volume was 20 μl. For amplification of a moderate GC-content fragment, 1 μl of pBluescript II KS (-) plasmid (GenBank: X52329.1) at 0.1 ng/μl was used as the template, with primers M13 forward (GTAAAACGACGGCCAG) and M13 reverse (CAGGAAACAGCTATGAC) at 0.4 μM each. The product was a 197 bp fragment with a GC-content of 53.8%. For GC-rich fragments amplification, plasmid containing the mouse Olig2 gene (0.1 ng/μl) was used as the template. With primers Olig2Qf1 (CACAGGAGGGACTGTGTCCT) and Olig2Qr1 (GAGGAGGTGCTGGAGGAAG) at 0.4 μM each, a 150 bp fragment with a GC-content of 68.0% (high GC) was amplified. For super high GC-rich fragment amplification, primers Olig2Qf2 (AGATCTACGGGGGTCACCA) and Olig2Qr2 (GGTAGAGAGGCGCTGGACAC) at 0.4 μM each were used, yielding a 208 bp fragment with a GC-content of 78.4%. The PCR included initial denaturation at 95°C for 1 min, followed by 40 cycles of denaturation at 95°C for 15 sec, annealing at 52°C (moderate GC) or 60°C (GC-rich) for 30 sec, and extension at 72°C for 30 sec, with plate reading at the end of each cycle. A final melting curve analysis was performed by incrementally increasing the temperature from 72°C to 96°C in 0.1°C steps. Different PCR enhancers (Table 1) were added to the reaction at various concentrations, each condition was tested in triplicates.

### PCR conditions

To evaluate the impact of PCR enhancers on the thermostability and inhibitor resistance of Taq DNA polymerase while amplifying a target DNA fragment with moderate GC-content, we used 1 μl of pBluescript II KS (-) plasmid (0.1 ng/μl), 0.2 μM of M13 forward and reverse primers, 0.2 mM dNTPs, and 1 unit (U) of Taq DNA polymerase (Sangon Biotech CN#B500010) in a 20 μl reaction. For thermostability analysis, the Taq DNA polymerase was preheated at 95°C for either 15 min or 30 min with or without PCR enhancers. For inhibitor resistance analysis, 0.0023 U or 0.0047 U heparin (Sangon Biotech A603251) was added to the PCR reaction with or without PCR enhancers. PCR was performed under the following program: initial denaturation at 95°C for 3 min, followed by 35 cycles of denaturation at 95°C for 30 sec, annealing at 55°C for 15 sec, extension at 72°C for 30 sec, and a final extension at 72°C for 10 min.

To assess the impact of PCR enhancers on the inhibitor resistance of Taq DNA polymerase while amplifying a DNA fragment with high and super high GC-content, we used 1 μl of plasmid containing the mouse Olig2 gene (0.1 ng/μl) as the template, 0.4 μM of Olig2Qf1 and Olig2Qr1 or Olig2Qf2 and Olig2Qr2e primers, 0.2 mM dNTPs, and 1 U of Taq DNA polymerase (Sangon Biotech CN#B500010) in a 20 μl reaction. Depending on the GC content, either 0.0094 U or 0.0375 U heparin was added for high GC PCR, and 0.0047 U or 0.0186 U heparin was used for super high GC PCR. PCR was performed with the following program: initial denaturation at 95°C for 3 min; followed by 35 cycles of denaturation at 95°C for 30 sec, annealing at 60°C for 30 sec, extension at 72°C for 30 sec, and a final extension at 72°C for 10 min.

For the amplification of long fragments with moderate GC-content, we used 1 μl of λ phage DNA (GenBank NC_001416.1) (1 ng/μl) as the template, with 0.4 μM of each primers L52F (GCGTTTCCGTTCTTCTTCGTCATAACTT) and L10139R (CAAAACTCAGCTCACCGTCGAACACTT) or L15635R (CTTTTTAAGCCATCCACCGGACCTTC), and 1 U of Taq/Pfu DNA polymerase mixture (Taq/Pfu = 100/3). The amplification produced a 10 kb fragment with 56.5% GC-content (L52F and L10139R), and a 15 kb fragment with 57.0% GC-content (L52F and L15635R). PCR was performed with the following program: initial denaturation at 95°C for 3 min, followed by 22 or 26 cycles of denaturation at 95°C for 15 sec, annealing and extension at 66°C for 8 min, and a final extension at 72°C for 15 min.

To evaluate the impact of PCR enhancers on amplification of a GC-rich region-containing long DNA fragment, we used 1 μl of 1 ng/μl L-Cas9 plasmid as the template, with 0.25 μM of each primer LCas9-88F (CCGTGCATGCCGATTGGTGGAAGT) and LCas9-8166R (GGGCGGAGTTAGGGGCGGGATAGC). LA Taq (TaKaRa RR900/903) was used as the DNA polymerase. The amplification product was an 8 kb fragment with a moderate GC-content of 51.8%, which includes a highly GC-rich region (87 bp of 93.1% GC-content within a 407 bp region of 71.3% GC-content). The PCR was performed with the following program: initial denaturation at 98°C for 3 min, followed by 35 cycles of denaturation at 98°C for 15 sec, annealing and extension at 66°C for 8 min, and a final extension at 72°C for 15 min.

## Results

### Betaine, sucrose and trehalose showed promising results as PCR enhancers

**Fig 1:**
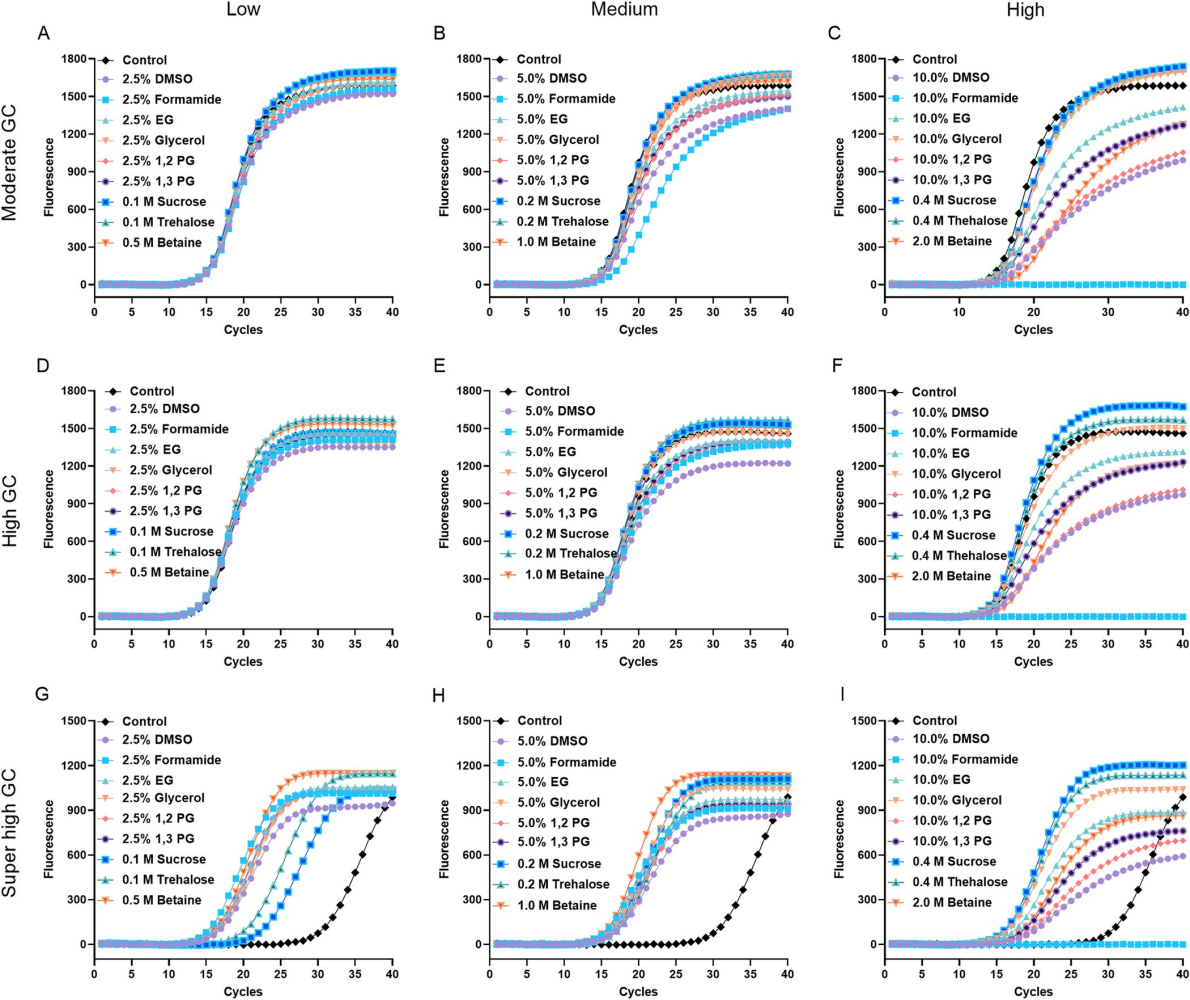
PCR enhancers have different effects in amplification of fragment with moderate, high and super high GC-content. Different PCR enhancers were tested at various conditions on the amplification efficiency by real-time PCR. (A-C) Representative amplification curve of the moderate GC fragment (53.8% GC), (D-F) high GC fragment (68.0% GC), and (G-I) super high GC fragment (78.4% GC).

We have selected nine different candidate PCR enhancers that can promote the amplification efficiency of GC-rich fragments. Since their effect in normal fragment amplification remains elusive, we tested their effect in amplification of a DNA fragment with moderate GC-content (53.8% GC) at various concentrations by real-time PCR (Fig 1 and Table 1). The results showed that all PCR enhancers, except trehalose and sucrose tested at their lowest concentration, reduced the amplification efficiency of moderate GC-content DNA fragments. This effect was more pronounced with higher concentrations of PCR enhancers, particularly formamide, which caused amplification failure at 10%. The results indicated that these PCR enhancers are inhibitory to the amplification of moderate GC-content DNA fragment (Fig 1A–1C and Table 1). Subsequently, we tested the effects of these enhancers in amplification of a high GC fragment (68.0% GC). The results showed that all the enhancers improved the PCR amplification efficiency slightly when used with low concentrations. When used at high concentrations, DMSO, 1,2-PG, 1,3-PG, EG, formamide, and betaine inhibited the PCR amplification efficiency. In contrast, glycerol, trehalose and sucrose enhanced amplification efficiency at all tested concentrations (Fig 1D–1F and Table 1). Finally, we used a DNA fragment of super high GC content (78.4% GC) to test the effects of enhancers on amplification. Compared with control, all enhancers were able to improve the PCR amplification efficiency. Notably, all those enhancers except trehalose and sucrose exhibited more pronounced promoting effects when used at low- and medium-concentrations than at high concentrations (Fig 1G–1I and Table 1). Overall, these enhancers could increase the amplification of high GC fragment at appropriate concentrations. However, they will inhibit the amplification of fragment with moderate GC content, especially used at high concentrations. Note that, 10% formamide used in this study prevented the amplification of any of the tested fragments. This is supported by previous research showing that formamide should not be used at concentration exceeding 10% [12, 18].

Compared with control group, all the tested PCR enhancers significantly lowered the melting temperature (Tm) of PCR products (Table 1). DMSO, formamide and 1,2-PG most significantly reduced the Tm value, decreasing it by more than 6°C per 10% for the DNA fragment with GC-contents ranging from moderate to super high. 2 M of betaine reduced Tm value of 4.5°C, 6.0°C and 6.3°C for the DNA fragments with moderate, high and super high GC-content, respectively. Note that betaine is a zwitterion that reduced the third hydrogen bond of G-C base pair, it is thus more effective in amplification of GC-rich DNA fragment [10, 19]. EG and 1,3-PG are slightly weaker than DMSO, formamide and 1,2-PG but stronger than glycerol in reducing Tm value. Sucrose and trehalose showed similar effect that they both reduce the Tm value or more than 2°C at 0.4 M for the DNA fragment with various GC-contents.

### Betaine, sucrose and trehalose greatly enhanced the thermostability of Taq DNA polymerase

**Fig 2:**
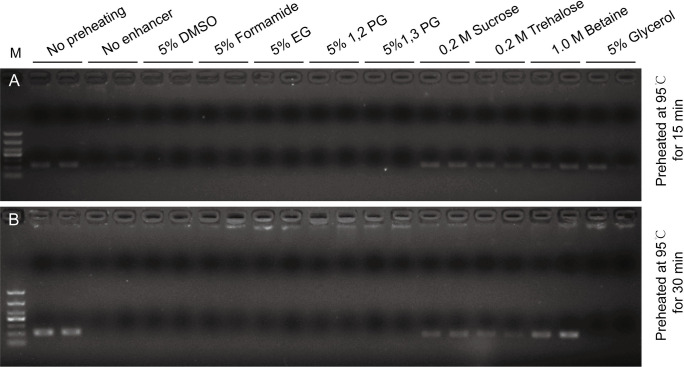
Enhancers such as betaine, sucrose and trehalose increase the thermostability of Taq DNA polymerase, but some enhancers function conversely. Different PCR enhancers were used to test their effect on the thermostability of Taq DNA polymerase. Before subjected to thermocycling, PCR reaction mixtures were preheated at 95°C for 15 min (A) or 30 min (B). M: TaKaRa DL1000 DNA Marker. No preheating: PCR mixtures were not preheated. No enhancer: PCR mixtures without enhancers. All conditions were tested in duplicate. The original gel images are available in the S1 Raw images.

To evaluate the effect of these PCR enhancers on the thermostability of Taq DNA polymerase, we preheated the PCR mixtures at 95°C for either 15 min or 30 min, with or without PCR enhancers, before subjected to thermocycling. Compared with the no preheating group, Taq DNA polymerase was partially heat-inactivated when preheated for 15 min at 95°C, resulting decreased amplification efficiency (Fig 2A). There were no visible bands when Taq DNA polymerase was preheated for 15 min with enhancers such as DMSO, formamide, EG, 1,2-PG and 1,3-PG (Fig 2A), suggesting that these PCR enhancers decreased the thermostability of Taq DNA polymerase. In contrast, when PCR enhancers such as sucrose, trehalose, betaine and glycerol were used during the 15 min preheating at 95°C, the amplification efficiencies are comparable to positive control, indicating that they protect Taq DNA polymerase from heat-inactivation.

When the PCR mixtures were preheated at 95°C for 30 min (Fig 2B), glycerol was insufficient to thermostabilize Taq DNA polymerase, as the amplification band disappeared. However, sucrose and trehalose still thermostabilized Taq DNA polymerase, with amplification bands clearly visible, albeit slightly weaker than in the no preheating group. Notably, the band of PCR products with betaine as an enhancer was comparable with the positive control, indicating a strong thermostabilizing effect of betaine on Taq DNA polymerase. These results demonstrated that betaine is the most effective enhancer at thermostabilizing Taq DNA polymerase, followed by sucrose and trehalose, and glycerol being the least effective.

### Betaine showed the highest resistance to the PCR inhibitor heparin

**Fig 3:**
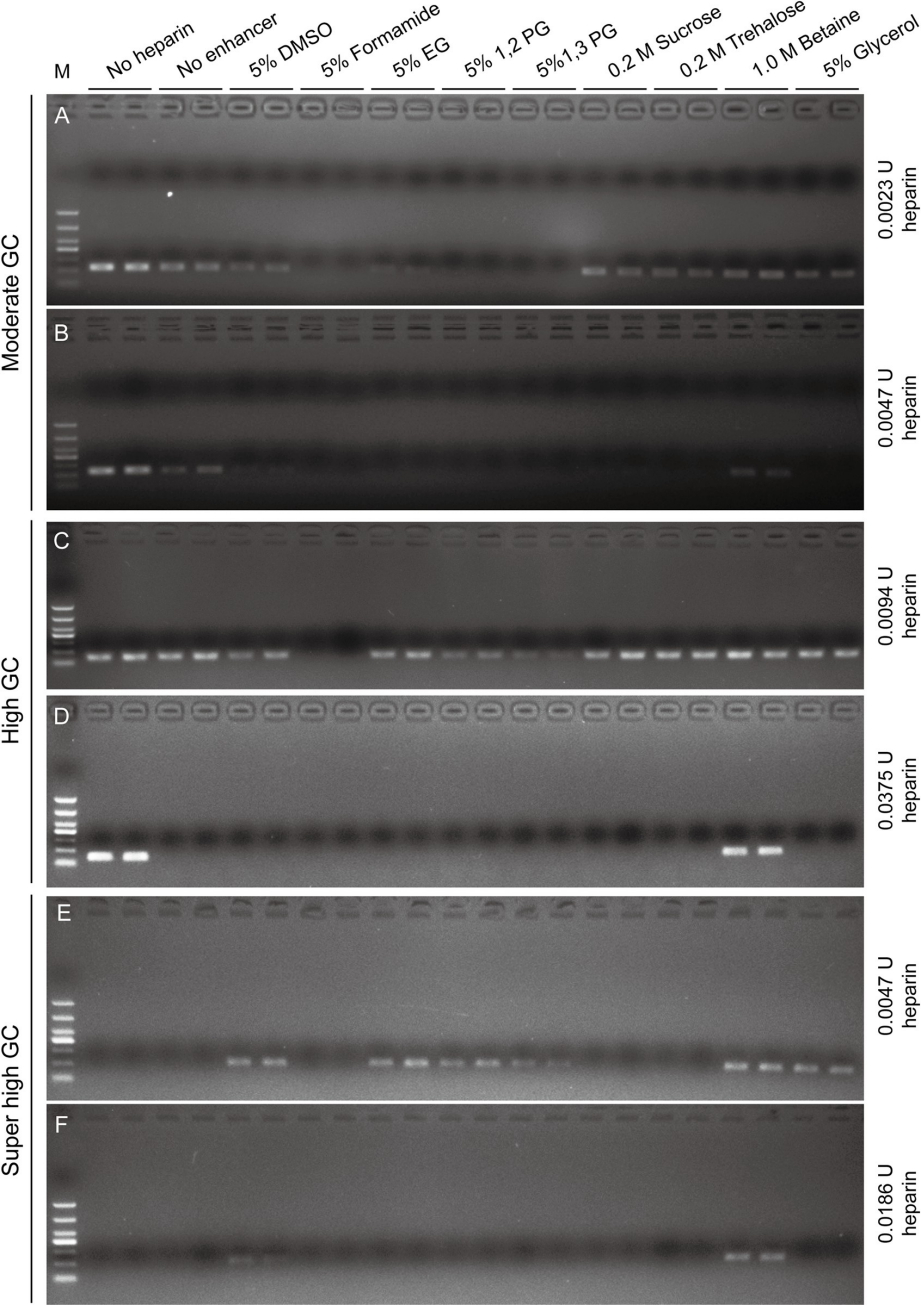
Analysis of the resistance effect of Taq DNA polymerase to PCR inhibitor with PCR enhancers. DNA fragments with moderate, high and super high GC-content were used for the analysis. (A) 0.0023 U heparin or (B) 0.0047 U heparin was added to the 20 μl PCR reaction with different PCR enhancers to amplify the moderate GC fragment. (C) 0.0094 U heparin or (D) 0.0375 U heparin was added to the 20 μl PCR reaction with different PCR enhancers to amplify the high GC fragment. (E) 0.0047 U heparin or (F) 0.0186U heparin was added to the 20 μl PCR reaction with different PCR enhancers to amplify the super high GC fragment. No heparin: PCR mixtures without addition of heparin. No enhancer: PCR mixtures without enhancers. All conditions were tested in duplicate. The original gel images are available in the S1 Raw images.

To examined the resistance effect of PCR enhancers to inhibitor, we used heparin as a Taq DNA polymerase inhibitor. For the moderate GC DNA fragment amplification (Fig 1 and Table 1), PCR products were weaker when 0.0023 U of heparin was included in the reaction, compared with the no heparin group. The target bands were invisible when PCR enhancers such as formamide, 1,2-PG and 1,3-PG were added, indicating that they significantly inhibit PCR amplification. The target bands with DMSO and EG were slightly weaker than those from no enhancer group, suggesting some inhibitory effects. The target bands of PCR with sucrose, trehalose, betaine and glycerol were similar to those of no enhancer group (Fig 3A). However, when heparin concentrations were increased, the target bands in DMSO and sucrose groups became barely visible, with bright bands present only in the betaine group (Fig 3B). To test if these PCR enhancers have different effect on the amplification of various templates, we then used a high GC-content DNA fragment for amplification (Fig 1 and Table 1). Unlike moderate GC fragment amplification, PCR efficiency was not significantly inhibited by adding 0.0094 U of heparin. However, PCR products were weaker with PCR enhancers such as DMSO, 1,2-PG and 1,3-PG, and invisible with formamide (Fig 3C). When heparin was increased to 0.0375 U, amplifications were detectable only in the no heparin and betaine groups, suggesting betaine has strong resistance to heparin (Fig 3D). We further test these PCR enhancers with a super high GC content DNA fragment. Since it is difficult to amply the super high GC fragment without PCR enhancers (Fig 1 and Table 1), the PCR products were invisible in the no heparin and no enhancer groups (Fig 1E and 1F). However, target PCR products were clearly visible with the tested PCR enhancers, except for formamide, sucrose and trehalose, suggesting that they enhanced the amplification of GC-rich fragment in the presence of heparin (Fig 3E). Even with 0.0186 U of heparin, betaine and DMSO still enhanced the PCR amplification of GC-rich DNA fragment (Fig 3F). Taken together, these results suggested that betaine has a great resistance ability to heparin, while certain PCR enhancers, especially formamide, inhibit PCR in the presence of heparin.

### Betaine exhibited an opposite effect in PCR amplification of long fragments with or without GC-rich region

**Fig 4:**
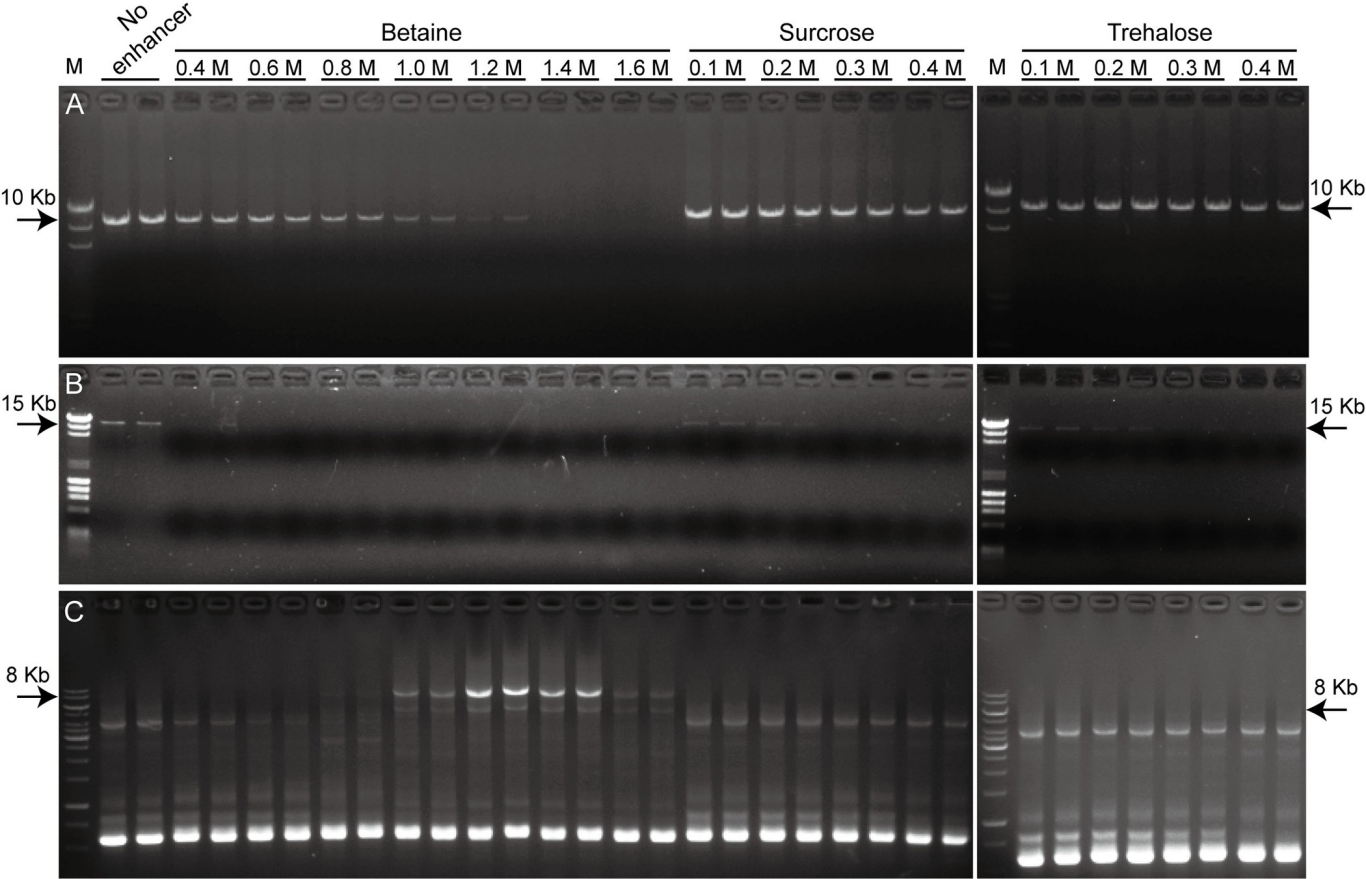
Dose dependent effect of betaine in PCR amplification of long fragment with or without GC-rich region. Various concentrations of betaine, sucrose and trehalose were tested for the amplification of a 10 kb (A) and a 15 kb λ phage DNA fragment (B). An 8 kb long DNA fragment containing a GC-rich region was subjected to amplification (C). All conditions were tested in duplicate. The original gel images are available in the S1 Raw images.

We further tested betaine, sucrose and trehalose in the amplification of long DNA fragments, as they outperformed other tested enhancers in thermostability, GC-rich amplification and resistance of PCR inhibitor. We firstly tested these PCR enhancers to amplify a 10 kb fragment with 56.5% GC-content. The results showed that the yield of PCR products decreased with increasing concentrations of betaine. Amplification bands disappeared when betaine was used at concentration higher than 1.4 M. In contrary, sucrose and trehalose have minimal negative effect on the amplification of the 10 kb fragment (Fig 4A). We next tested the amplification of a 15 kb fragment with 57.0% GC-content. The results showed that no specific band were observed when betaine was used. We detected relatively weaker amplification of the 15 kb fragment with 0.1 M to 0.2 M sucrose and trehalose compared to the no enhancer group (Fig 4B). These results indicate that, rather than promoting the amplification of long fragments with moderate GC-content, high concentrations of PCR enhancer may inhibit amplification efficiency. We then tested these enhancers for the PCR amplification of a long fragment containing a GC-rich region from LCas9 plasmid, which was previously demonstrated difficult to be amplified by PCR [16]. The entire fragment has moderate GC-content (51.8%), but contains a highly GC-rich region (an 87 bp segment with 93.1% GC-content within a 407 bp segment with 71.3% GC-content). The results showed that the specific fragment could not be amplified without PCR enhancers. While sucrose and trehalose had little effect, specific amplification was detectable when betaine was used at the concentration of 0.8 M, peaked at 1.2 M and declined at 1.6 M. These results indicate that PCR enhancers, especially betaine, are required for PCR amplification of long fragments containing GC-rich regions (Fig 4C).

### Betaine and sucrose synergistically promote amplification of GC-rich region-containing long DNA fragments

**Fig 5:**
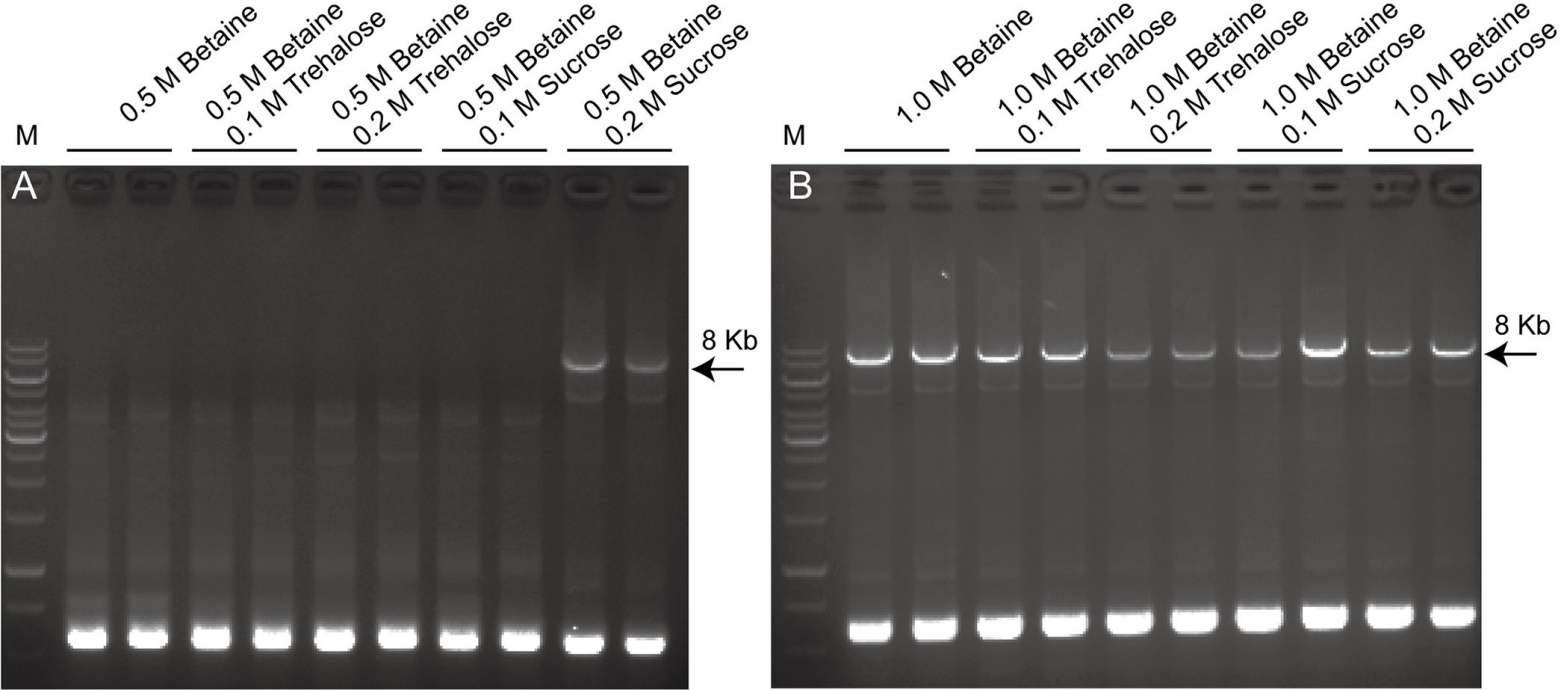
Synergistic effect of betaine and sucrose in promote amplification of GC-rich region-containing long DNA fragment. (A) 0.5 M betaine combined with different concentrations of trehalose and sucrose as PCR enhancers. (B) 1 M betaine plus different concentrations of trehalose and sucrose were used as PCR enhancers. All conditions were tested in duplicate. The original gel images are available in the S1 Raw images.

To determine optimal conditions for PCR enhancers to promote the amplification of long and GC-rich fragments while minimizing their inhibitory effects on normal fragments, we compared several different combinations of betaine with sucrose and trehalose. We tested the PCR enhancers to amplify the 8 kb fragment from LCas9 plasmid. The results showed that specific amplification bands were detectable with 0.5 M betaine plus 0.2 M sucrose (Fig 5A). Combinations of 1 M betaine with 0.1 M to 0.2 M sucrose or trehalose were also effective in amplifying the specific GC-rich fragment (Fig 5B). These results indicated that 0.5 M betaine plus 0.2 M sucrose or 1 M betaine plus 0.1 M sucrose can effectively promote the amplification of GC-rich fragments while keeping their negative effects on normal fragment amplification to a minimum.

## Discussion

Since the advent of PCR technology in the 1980s, scientists have been committed to enhancing its amplification efficiency through various means, especially for some complex PCR amplification processes [1, 2]. Among them, an effective method is to add PCR enhancers, which can significantly optimize the PCR reaction and improve the efficiency and specificity of amplification. Numerous PCR enhancers are characterized by their ability to thermostabilize DNA polymerase, increase its tolerance to inhibitors, or decrease the melting temperature of GC-rich DNA fragments. These enhancers have been successfully used to improve the PCR amplification efficiency and specificity of targets under various conditions [9–13, 15, 17]. However, there is still a lack of systematic analysis regarding the conditions under which PCR enhancers are beneficial.

In this study, we selected nine different PCR enhancers previously reported to increase PCR amplification efficiency [7–17]. We found that appropriate concentrations of enhancers can significantly improve PCR amplification efficiency for GC-rich fragments. However, excessively high concentrations, such as 10% formamide, led to amplification failure for all tested targets (Fig 1 and Table 1). This is consistent with previous studies indicating that high concentrations of PCR enhancers like 0.6 M trehalose, 2 M betaine, 10% formamide inhibit PCR amplification efficiency of GC-rich fragments [9, 13, 18]. Notably, all tested PCR enhancers exhibited some levels of inhibitory effect for amplification of DNA fragment with moderate GC-content even at low concentrations (Table 1, Figs 1 and 4). The inhibition effect of these PCR enhancers may be caused by that: (1) decreased activity of Taq DNA polymerase in the presence of PCR enhancers, as betaine, 1,2-PG and formamide decreased the extension rates of Taq DNA polymerase with increasing concentrations [18]; (2) high concentrations of PCR enhancer significantly lowered the Tm of DNA fragment, thus reducing the primer-template annealing efficiency; and (3) PCR enhancers such as DMSO, formamide, 1,2-PG, 1,3-PG and EG greatly reduced the thermostability of Taq DNA polymerase (Fig 2).

PCR enhancers such as betaine, sucrose, trehalose and glycerol increased the thermostability of Taq DNA polymerase (Fig 2). Our results showed that 1 M betaine showed the best thermostabilizing effect on Taq DNA polymerase, which is different from a previous study where 0.2 M trehalose showed a greater thermostabilizing effect than 1.2 M betaine [13]. This discrepancy may be due to 1.2 M betaine’s significant inhibitory effect on amplifying DNA fragments of moderate GC-content [18], as observed in our results (Figs 1A and 4B), thus reducing amplification efficiency. Additionally, not all PCR enhancers increased the thermostability of Taq DNA polymerase. For example, 5% of DMSO, formamide, 1,2-PG, 1,3-PG and EG destabilized Taq DNA polymerase and caused PCR failure when Taq DNA polymerase was preheated at 95°C for 15 min (Fig 2). Chemically modified Taq DNA polymerase, which is inactive at room temperature and requires activation at 95°C for 10 min, is widely used in hot start PCR to minimize non-specific amplification. These PCR enhancers that destabilized Taq DNA polymerase may cause substantial loss of activity during the heat activation of chemically modified Taq DNA polymerase, thereby significantly reducing PCR amplification efficiency.

Interestingly, only betaine demonstrated clear resistance to heparin as a PCR inhibitor across all tested conditions. For DNA fragments with moderate GC-content, all other tested PCR enhancers reduced the amplification products in the presence of heparin (Fig 3A and 3B). Same trends were observed for high GC-content fragments (Fig 3C and 3D). In contrast, for super high GC-content fragments, most PCR enhancers increased amplification products (Fig 3E and 3F). The improved amplification of super high GC-content fragment could be due to enhanced amplification of GC-rich DNA fragments by these PCR enhancers, supported by a previous study showing that 1,2-PG improves PCR amplification of a GC-rich fragment in the presence of blood [17]. Taken together, these results suggested that apart from betaine, the other tested PCR enhancers did not show resistance to heparin (Fig 3).

Although betaine alone suffices for amplification of long DNA fragments with GC-rich regions (Fig 4), effective conditions can be slightly inhibitory for DNA fragment with moderate GC-content (Table 1, Figs 1 and 4). The combination of betaine with DMSO or trehalose has been successfully used for amplification of GC-rich DNA fragments [3, 16, 17, 20]. It is interesting that long DNA fragment with GC-rich region can also be amplified using 0.5 M betaine combined with 0.2 M sucrose or trehalose (Fig 5). Since sucrose and trehalose showed the mildest inhibitory effects even at high concentrations (Table 1 and Fig 1), and previous study showed that betaine below 0.5 M does not significantly decrease Taq DNA polymerase activity [18], the inhibitory effect on standard PCR is likely minimal. Sucrose serves as a good alternative to trehalose due to its lower cost and greater solubility, as our results showed that sucrose and trehalose had similar effects across all tests, while trehalose often formed aggregates in mixtures of 2.5 M betaine and 1 M trehalose stock solutions, which are difficult to re-dissolve.

In summary, betaine offers significant advantages among the tested enhancers for evaluating PCR amplification efficiency, thermostabilizing DNA polymerase, tolerance to PCR inhibitors, and amplifying long and GC-rich fragments. The combination of betaine and sucrose, both of which are sweet, can effectively manage various PCR amplification conditions.

## Supporting information

S1 Raw images (PDF)
