# Supplementary material for: Sweet enhancers of polymerase chain reaction
Source: PLoS One. 2024 Oct 29;19(10):e0311939. doi: 10.1371/journal.pone.0311939 (PMC11521273; doi:10.1371/journal.pone.0311939)
Supplement: S1 Raw images — (PDF) [file pone.0311939.s001.pdf]

Raw gel electrophoresis images corresponding to the Figures 3-5 in the paper.

Fig 2A

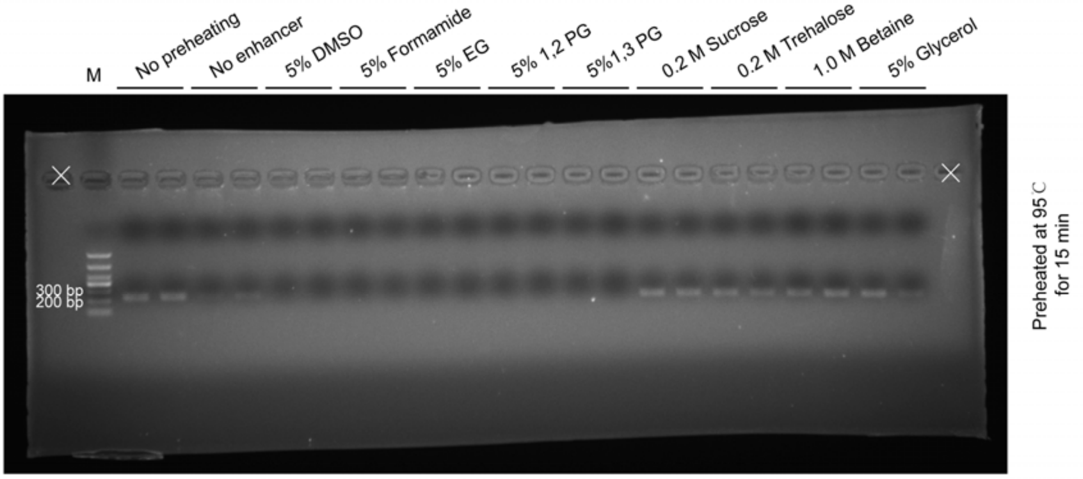

Fig 2B

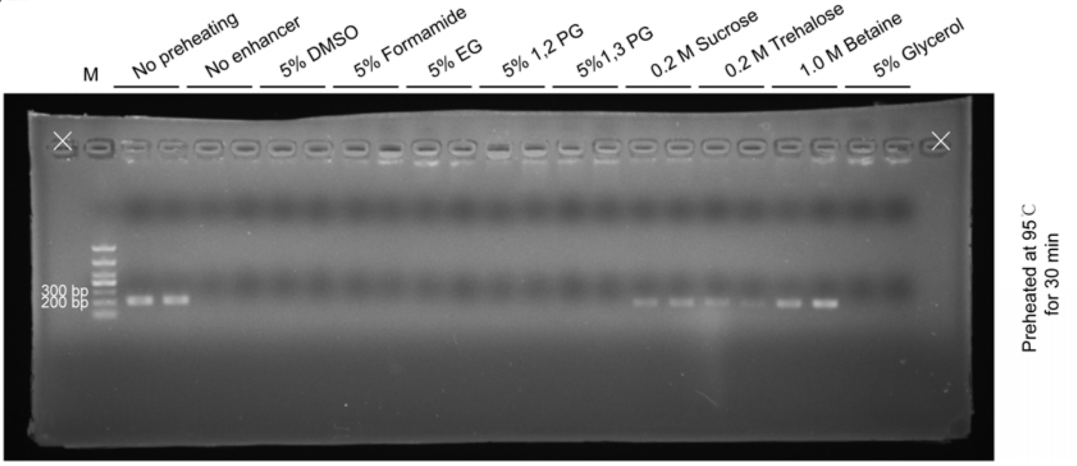

Fig 3A

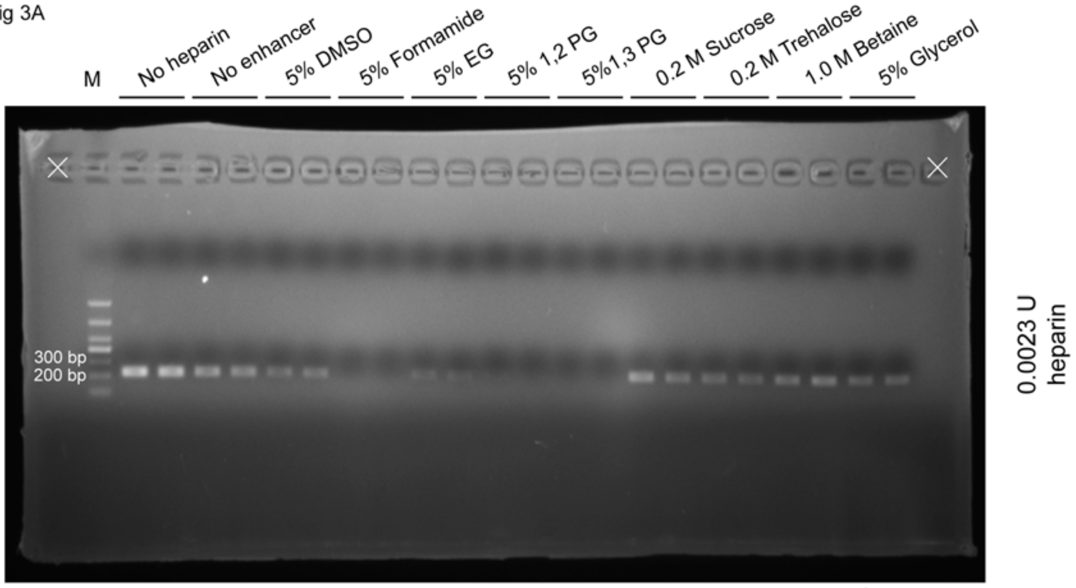
M
No heparin
No enhancer
5% DMSO
5% Formamide
5% EG
5% 1,2 PG
5%1,3 PG
0.2 M Sucrose
0.2 M Trehalose
1.0 M Betaine
5% Glycerol
300 bp
200 bp
0.0023 U heparin

Fig 3B

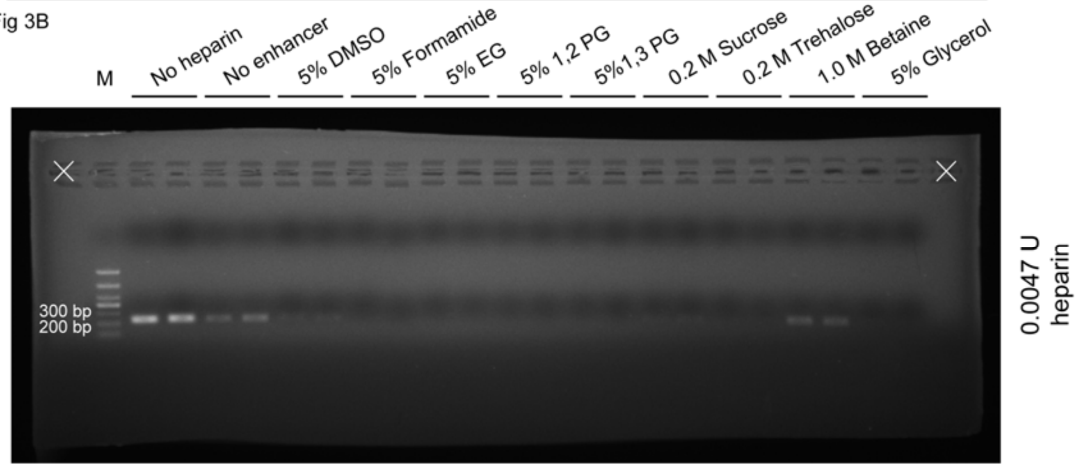
M
No heparin
No enhancer
5% DMSO
5% Formamide
5% EG
5% 1,2 PG
5%1,3 PG
0.2 M Sucrose
0.2 M Trehalose
1.0 M Betaine
5% Glycerol
300 bp
200 bp
0.0047 U heparin

Fig 3C

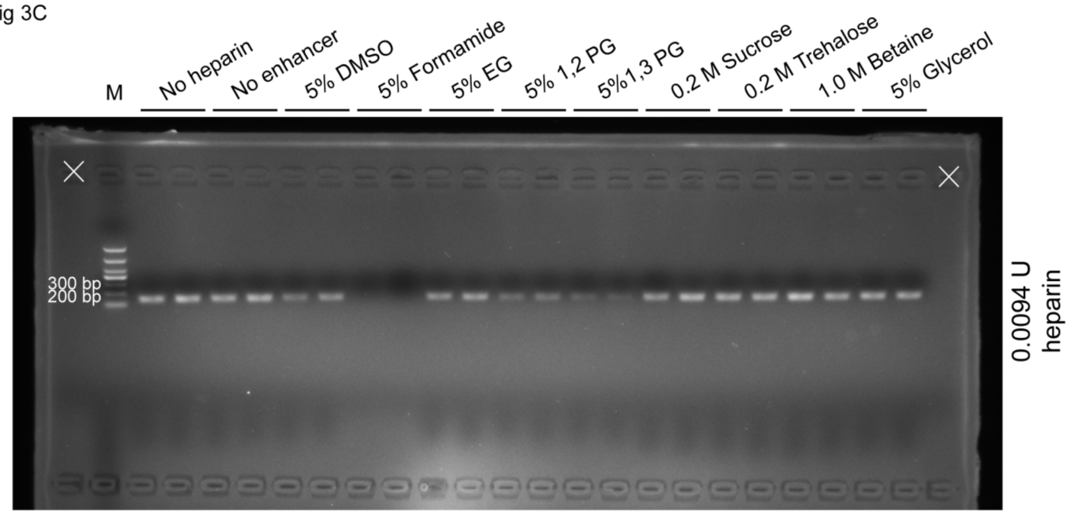
M
No heparin
No enhancer
5% DMSO
5% Formamide
5% EG
5% 1,2 PG
5%1,3 PG
0.2 M Sucrose
0.2 M Trehalose
1.0 M Betaine
5% Glycerol
300 bp
200 bp
0.0094 U heparin

Fig 3D

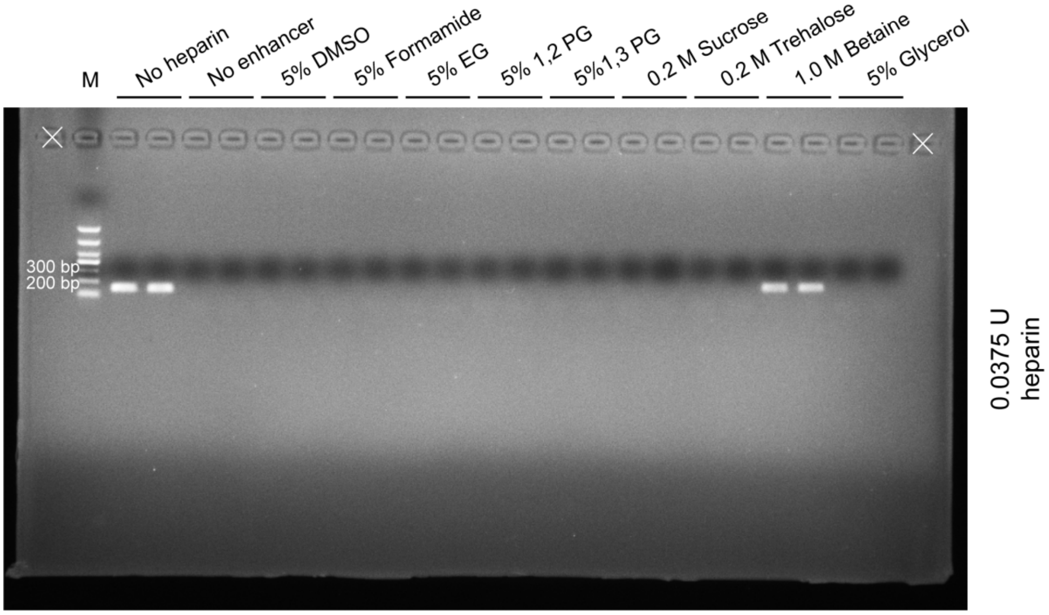
M
No heparin
No enhancer
5% DMSO
5% Formamide
5% EG
5% 1,2 PG
5%1,3 PG
0.2 M Sucrose
0.2 M Trehalose
1.0 M Betaine
5% Glycerol
300 bp
200 bp
0.0375 U heparin

Fig 3E

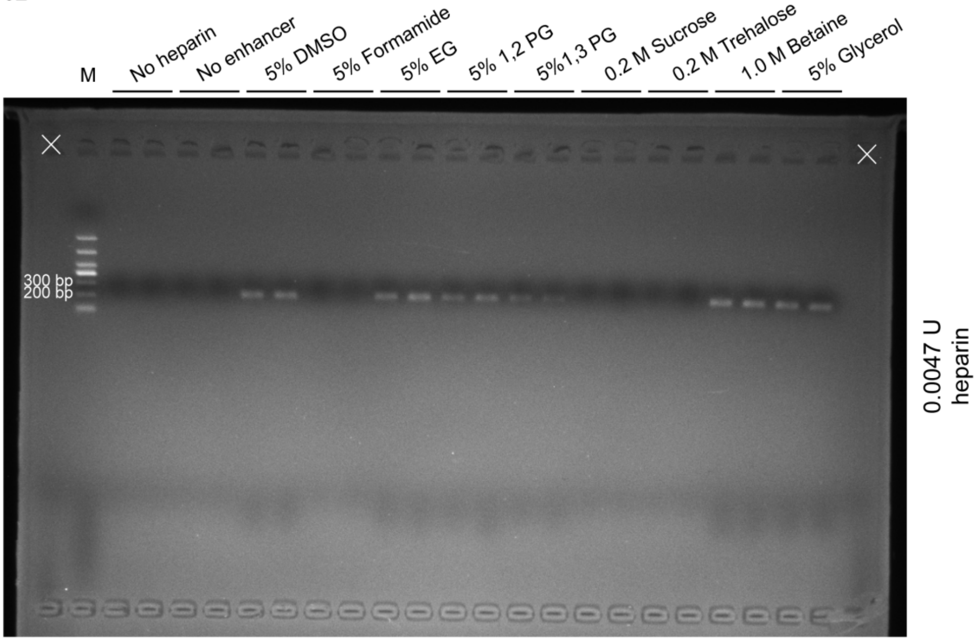
M
No heparin
No enhancer
5% DMSO
5% Formamide
5% EG
5% 1,2 PG
5%1,3 PG
0.2 M Sucrose
0.2 M Trehalose
1.0 M Betaine
5% Glycerol
300 bp
200 bp
0.0047 U heparin

Fig 3F

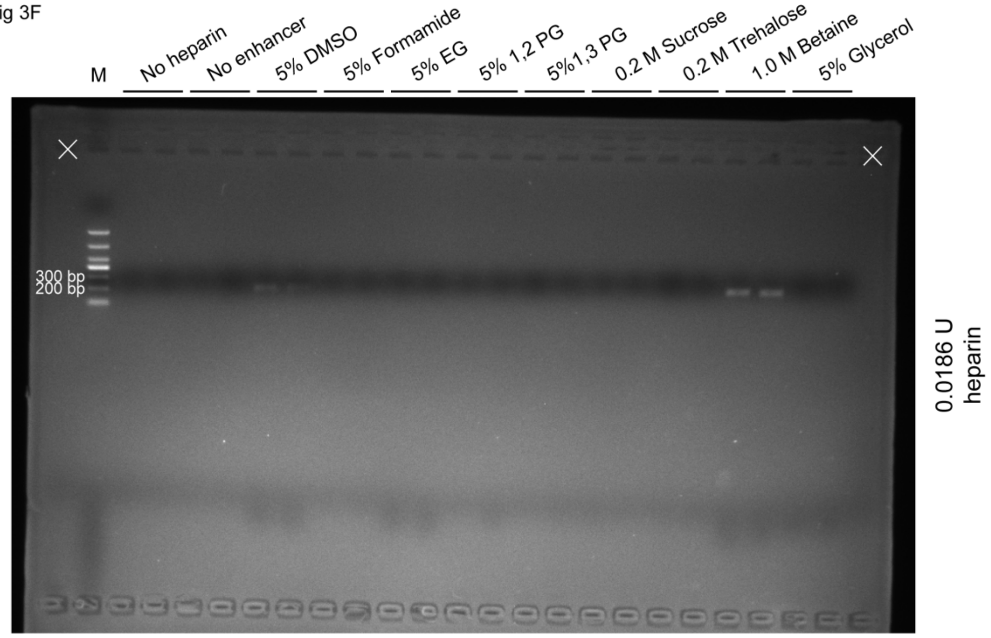
M
No heparin
No enhancer
5% DMSO
5% Formamide
5% EG
5% 1,2 PG
5%1,3 PG
0.2 M Sucrose
0.2 M Trehalose
1.0 M Betaine
5% Glycerol
300 bp
200 bp
0.0186 U heparin

Fig 4A

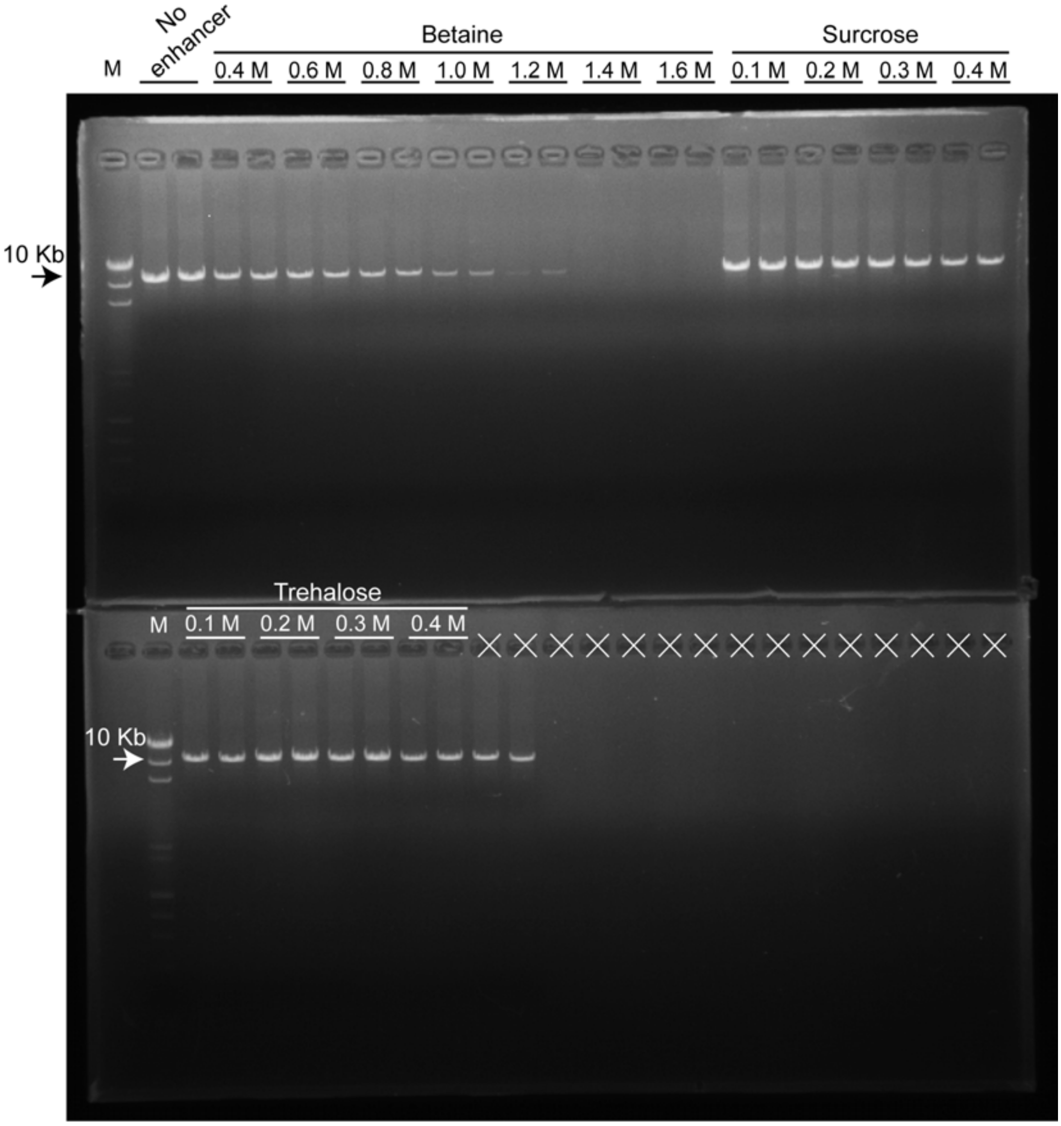
M
No enhancer
Betaine
0.4 M
0.6 M
0.8 M
1.0 M
1.2 M
1.4 M
1.6 M
Surcrose
0.1 M
0.2 M
0.3 M
0.4 M
10 Kb
Trehalose
M
0.1 M
0.2 M
0.3 M
0.4 M
10 Kb

Fig 4B

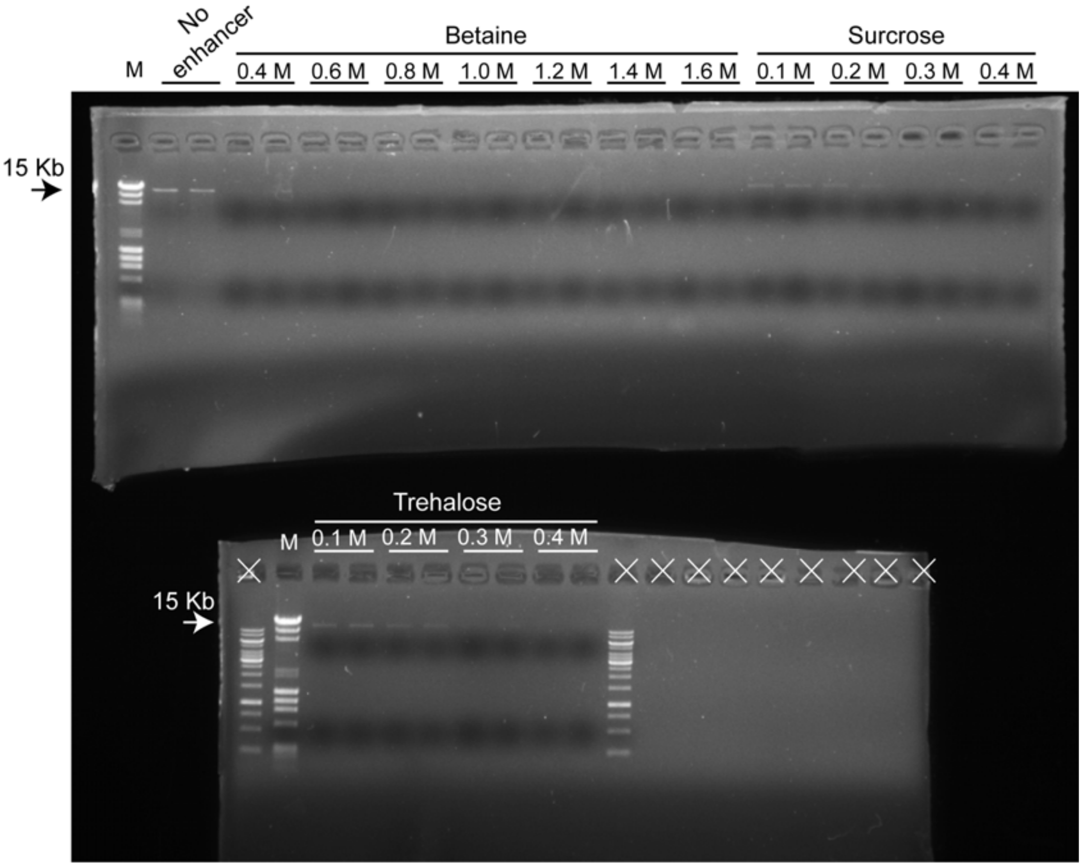

No enhancer
Betaine
Surcrose
M
0.4 M
0.6 M
0.8 M
1.0 M
1.2 M
1.4 M
1.6 M
0.1 M
0.2 M
0.3 M
0.4 M
15 Kb
Trehalose
M
0.1 M
0.2 M
0.3 M
0.4 M
15 Kb

Fig 4C

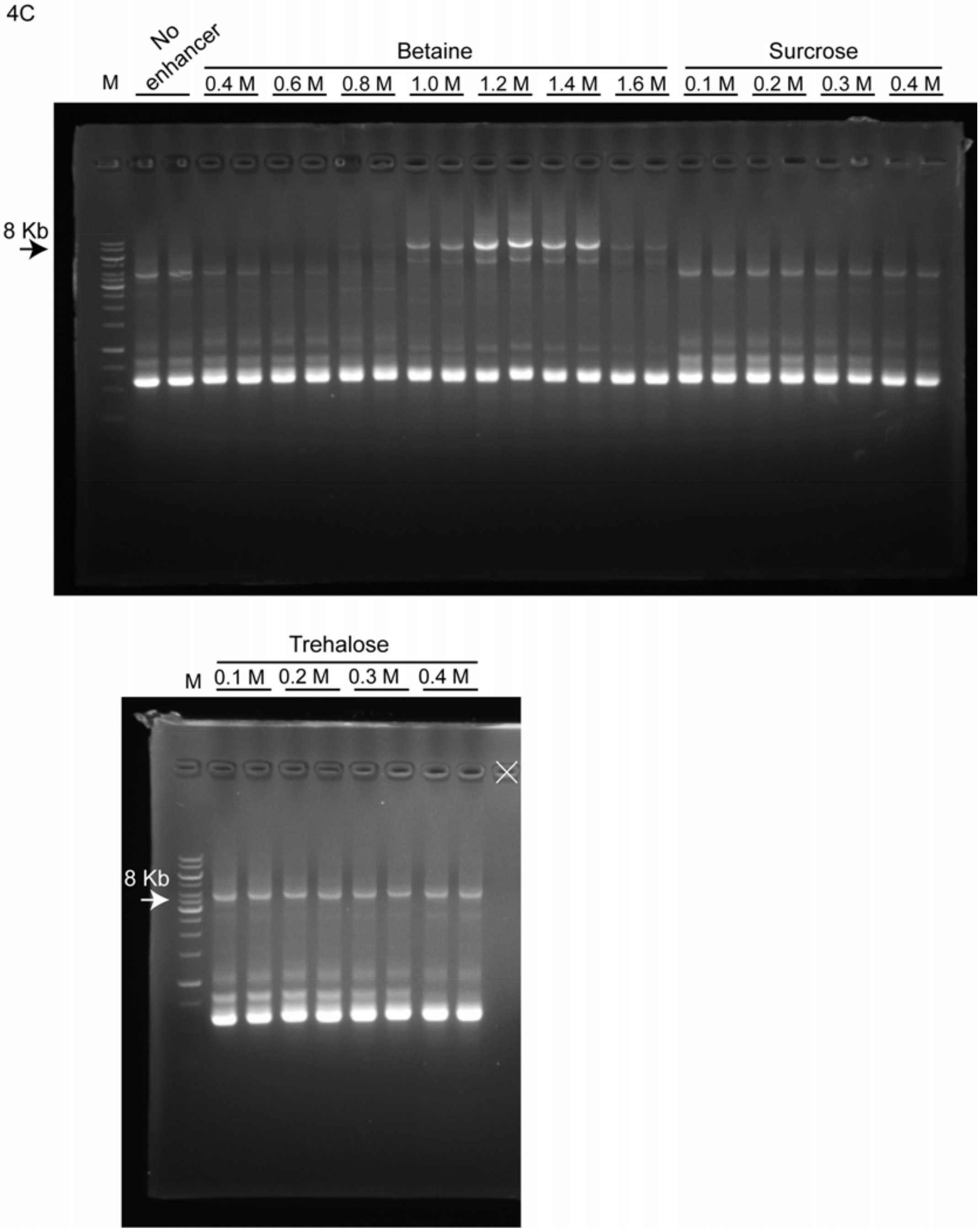

No enhancer
Betaine
Surcrose
M
0.4 M
0.6 M
0.8 M
1.0 M
1.2 M
1.4 M
1.6 M
0.1 M
0.2 M
0.3 M
0.4 M
8 Kb
Trehalose
M
0.1 M
0.2 M
0.3 M
0.4 M
8 Kb

Fig 5A

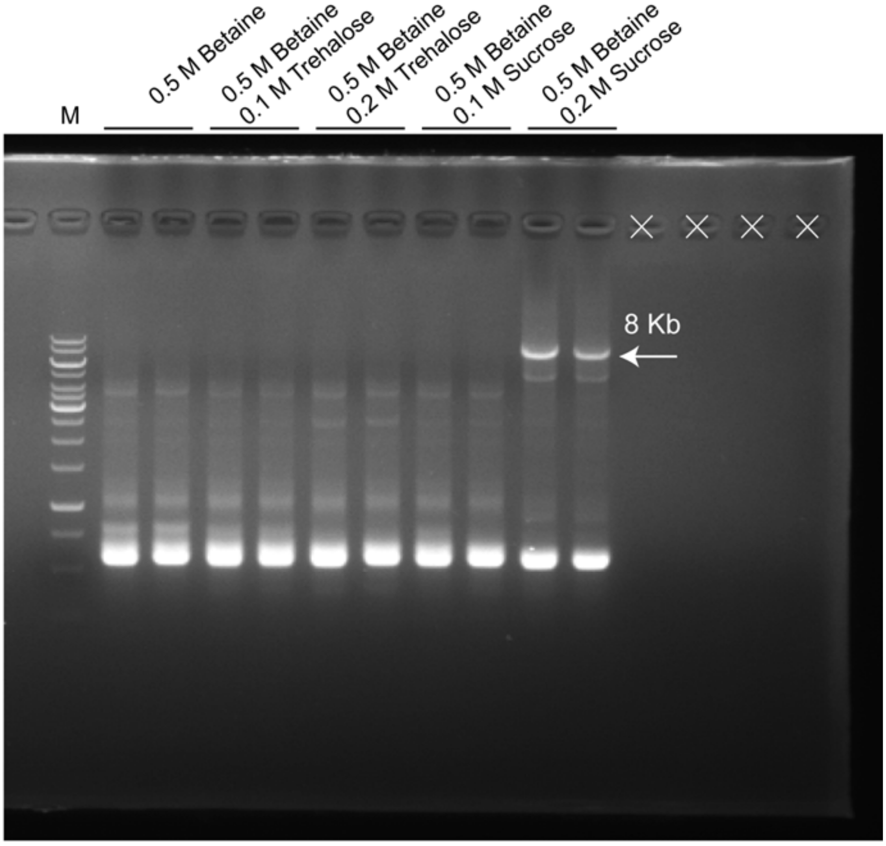
M
0.5 M Betaine
0.5 M Betaine
0.1 M Trehalose
0.5 M Betaine
0.2 M Trehalose
0.5 M Betaine
0.1 M Sucrose
0.5 M Betaine
0.2 M Sucrose
8 Kb

Fig 5B

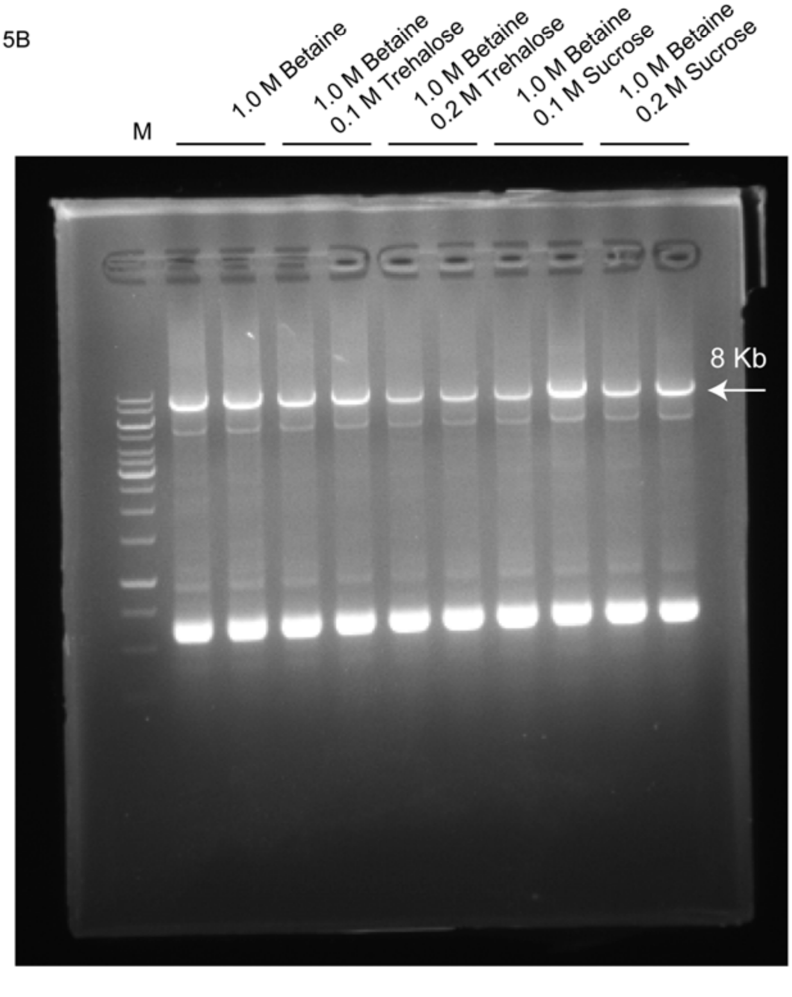
M
1.0 M Betaine
1.0 M Betaine
0.1 M Trehalose
1.0 M Betaine
0.2 M Trehalose
1.0 M Betaine
0.1 M Sucrose
1.0 M Betaine
0.2 M Sucrose
8 Kb
